# Precise Synthesis and Thin Film Self-Assembly of PLLA-*b*-PS Bottlebrush Block Copolymers

**DOI:** 10.3390/molecules26051412

**Published:** 2021-03-05

**Authors:** Eunkyung Ji, Cian Cummins, Guillaume Fleury

**Affiliations:** Laboratoire de Chimie des Polymères Organiques, Université de Bordeaux, CNRS, Bordeaux INP, LCPO, UMR 5629, F-33600 Pessac, France; eunkyungji@gmail.com (E.J.); c.cummins1988@gmail.com (C.C.)

**Keywords:** bottlebrush block copolymer, sequential ROMP, thin film self-assembly, nanoporous templates

## Abstract

The ability of bottlebrush block copolymers (BBCPs) to self-assemble into ordered large periodic structures could greatly expand the scope of photonic and membrane technologies. In this paper, we describe a two-step synthesis of poly(l-lactide)-*b*-polystyrene (PLLA-*b*-PS) BBCPs and their rapid thin-film self-assembly. PLLA chains were grown from *exo*-5-norbornene-2-methanol via ring-opening polymerization (ROP) of l-lactide to produce norbornene-terminated PLLA. Norbonene-terminated PS was prepared using anionic polymerization followed by a termination reaction with *exo*-5-norbornene-2-carbonyl chloride. PLLA-*b*-PS BBCPs were prepared from these two norbornenyl macromonomers by a one-pot sequential ring opening metathesis polymerization (ROMP). PLLA-*b*-PS BBCPs thin-films exhibited cylindrical and lamellar morphologies depending on the relative block volume fractions, with domain sizes of 46–58 nm and periodicities of 70–102 nm. Additionally, nanoporous templates were produced by the selective etching of PLLA blocks from ordered structures. The findings described in this work provide further insight into the controlled synthesis of BBCPs leading to various possible morphologies for applications requiring large periodicities. Moreover, the rapid thin film patterning strategy demonstrated (>5 min) highlights the advantages of using PLLA-*b*-PS BBCP materials beyond their linear BCP analogues in terms of both dimensions achievable and reduced processing time.

## 1. Introduction

Bottlebrush block copolymers (BBCPs) are densely grafted polymers with two or more different polymeric side chains attached to a linear polymeric backbone. The densely grafted side chains of BBCPs yield to an extended backbone conformation and thus reduce chain entanglement, which lowers kinetic barriers for self-assembly. Accordingly, BBCP systems undergo a rapid microphase separation for nanostructures exhibiting large periodicities beyond 100 nm. Due to these unique assets, BBCPs have received tremendous attention for photonic, surface coating, and drug delivery applications [1,2,3,4,5,6,7,8,9,10]. BBCPs are prepared either by grafting from, grafting to, or grafting through approaches [6,11,12]. Among these approaches, the grafting through method leverages the robust control of the macromolecular parameters, i.e., side chain length, backbone degree of polymerization, and grafting density. This route can afford 100% grafting density (one side chain per backbone repeating unit) using macromonomers (MMs) synthesized prior to the backbone polymerization. However, this route also suffers from the high viscosity of the reaction media and the steric hindrance linked to the bulky MMs [1,6].

To address these problems, ring opening metathesis polymerization (ROMP) of norbornenyl MMs can be used by taking advantage of both the release of ring strain energy from cyclic olefins upon ring opening, and the use of highly active ruthenium-based catalysts. Rajaram et al. demonstrated the polymerization of sterically hindered dendronized norbornenes using the highly active, fast-initiating third-generation Grubbs catalyst (G3) [13]. Subsequently, in 2009, Xia et al. reported the use of G3 for ROMP of MMs, producing various brush polymers with high molecular weights and a narrow dispersity [14]. They also demonstrated for the first time the polymerization of high molecular weight, narrowly dispersed brush block- and random- copolymers via sequential ROMP of MMs based on poly(*n*-butyl acrylate) (P*n*BA), poly(*t*-butyl acrylate) (P*t*BA), polystyrene (PS), and polylactide (PLA) [1].

Over the past decade, researchers have reported different synthetic routes to synthesize BBCPs via ROMP of norbornenyl MMs, and their self-assembly into ordered nanostructures with large domain sizes; however, only a few studies investigated BBCP morphological transitions depending on the volume fraction of the two blocks. Given the complex architecture of BBCPs, backbone and side chain lengths as well as block volume fractions need to be considered to establish a phase diagram as recently described by Zhulina et al. [15]. Runge et al. demonstrated how coil BBCPs could produce large cylindrical domains (100–200 nm), and the backbone chain length controlled domain sizes [16]. Subsequently, they reported the lamellar, cylindrical, or spherical morphologies depending on the BBCP chemical compositions [17]. Nevertheless, BBCP self-assembly produces lamellar morphologies over a large window of the complex phase diagram due to a highly extended backbone conformation [1,15,18,19,20,21].

To obtain non-lamellar morphologies, Jiang et al. introduced gradient interfaces between two blocks (PS and PLA): from PS-rich, followed by a gradated transition from PS to PLA, to PLA-rich along the backbone [22]. Side-chain symmetric gradient BBCPs exhibited cylindrical morphologies while their BBCP analogs arranged into lamellae. Additional efforts have been made to tune the BBCP morphologies by manipulating backbone or side chain asymmetry. For example, Gai et al. reported cylindrical morphologies from PS-*b*-PEO BBCPs at a high volume fraction of PEO (*f*_PEO_ ~ 0.8) [23]. Cho et al. demonstrated how asymmetric PS-*stat*-PLA bottlebrush statistical copolymers with longer PLA (5.6 kDa) and shorter PS (2.0 kDa) side chains exhibited cylindrical (at *f*_PS_ = 0.41–0.50) and lamellar (at *f*_PS_ = 0.67–0.70) morphologies [24]. Besides, lengthy annealing treatments are commonly employed for the promotion of the self-assembly in thin films [19]. To our knowledge, the best reported BBCP thin film study required 2–4 h of solvent annealing of a PS-*b*-PLA BBCP to produce well-ordered lamellar patterns [25]. Due to the complex interplay between architecture of BBCPs and self-assembly behavior in thin film, additional studies on microphase separated BBCPs are required to manipulate as well as understand BBCP thin film processing.

In this article, we report a route to synthesize BBCPs containing PLLA and PS, and detail their rapid self-assembly and morphological transitions depending on the PLLA block volume fraction using solvent vapor annealing. PLLA was chosen as one of polymeric side chains in our attempts to obtain various nanostructures taking advantage of the semi-crystallinity of PLLA [26,27,28]. In addition, selective etching of the PLLA block can create porous thin films, which can be used as sacrificial templates in the field of nanotechnology [29,30]. BBCPs were prepared by a two-step synthesis: (1) formation of MMs via anionic polymerization of styrene and ring opening polymerization of l-lactide using norbornene derivatives as an initiator and end-capping agents, respectively, and (2) sequential ROMP of the two MMs. PLLA volume fractions were controlled by adjusting the feed ratios between PLLA and PS MMs. PLLA-*b*-PS BBCP thin-films were then developed showing an impressive capacity to self-assemble with periods as large as 102 nm in the lamellar system described. Lastly, the BBCP thin-films were treated with oxygen plasma etching to form nanoporous templates.

## 2. Results and Discussion

### 2.1. Synthesis of Macromonomers

**Scheme 1:**
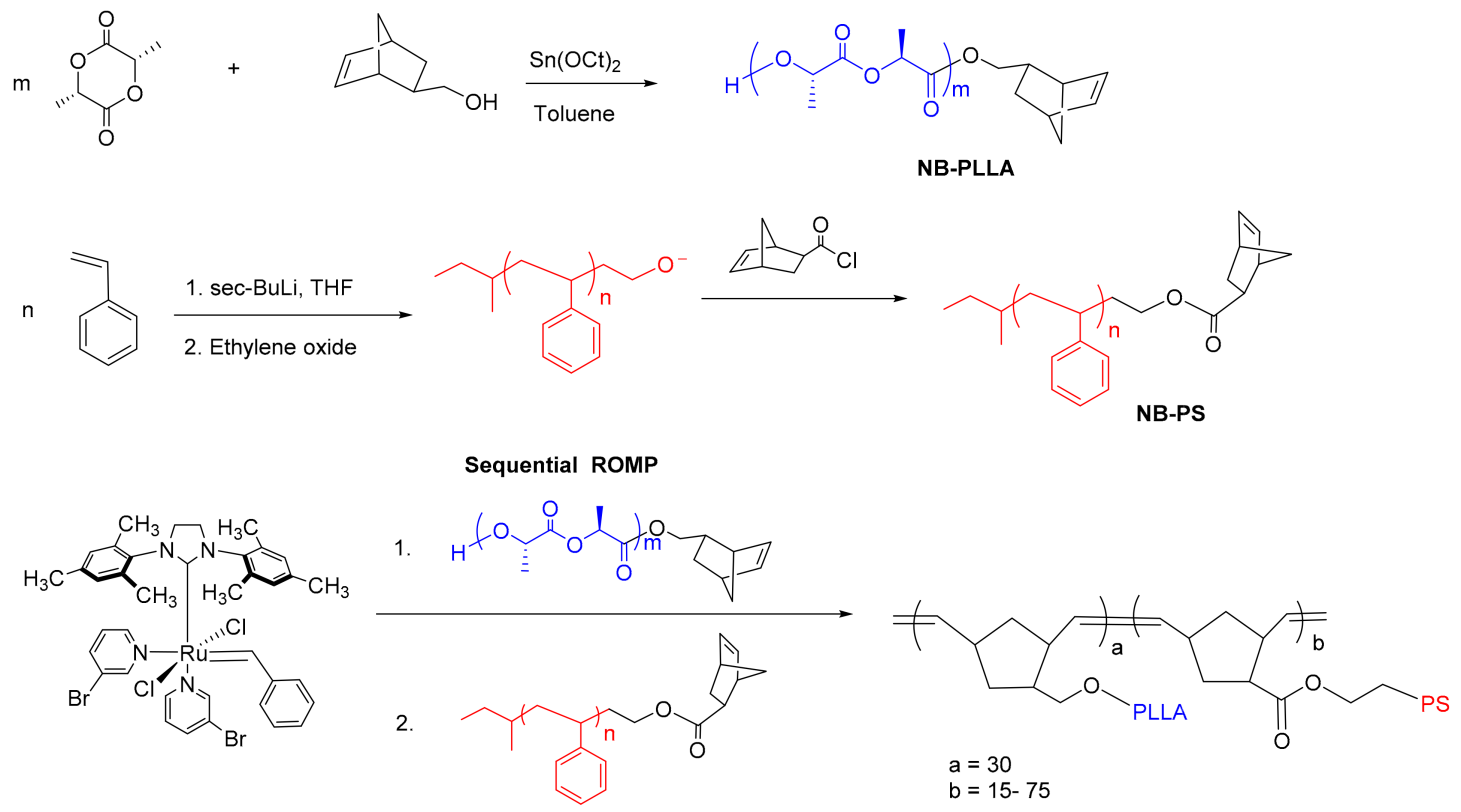
Synthetic scheme for the preparation of norbornene-functionalized MMs and sequential ROMP of MMs.

The desired norbornene-terminated MMs were prepared by a one-pot polymerization using norbornene derivatives as an initiator or an end-capping agent (see Scheme 1). Norbornenyl PLLA MM (NB-PLLA) was obtained by ring opening polymerization of l-lactide using *exo*-5-norbornene-2-methanol as an initiator. Norbornenyl PS MM (NB-PS) was synthesized by previously reported procedures: anionic polymerization of styrene, followed by reacting with ethylene oxide and then *exo*-5-norbornene-2-carbonyl chloride [31]. In particular, *exo*-norbornenes were used in this study because of their significantly higher reactivity than *endo*-isomers, arising from steric interactions between growing polymer chains and incoming monomers [14,32]. Molecular characteristics of these two MMs obtained by SEC, NMR, and MALDI-TOF MS are summarized in Table 1.

NMR spectroscopy confirms the complete attachment of norbornene end groups to PLLA and PS chains (Figures S1 and S2). In the ^1^H NMR spectra, the norbornenyl olefinic protons were observed at δ 6.08 ppm (NB-PLLA) and 6.02 ppm (NB-PS). ^13^C NMR spectra also exhibit two peaks at δ 136 and 137 ppm (NB-PLLA) and δ 136 and 138 ppm (NB-PS), which are attributed to the double bonds of norbornene. Furthermore, their molecular weights were determined from ^1^H NMR by comparing the integrations of the norbornenyl olefin (δ ~ 6 ppm) and polymer chain signals (δ 5.13–5.18 ppm for PLLA and δ 6.32–7.00 ppm for PS) (Table 1). For NB-PS, the calculated molecular weight was in good agreement with the one determined by SEC in THF using PS standards while an overestimated NB-PLLA molecular weight was observed by SEC.

MALDI-TOF MS analysis provides further verification of successful terminal functionalization with norbornene-derivatives. Figures S3 and S4 display MALDI-TOF spectra of NB-PLLA and NB-PS MMs. The peaks are separated by 72 and 104 mass units, corresponding to the molecular weights of a half of the lactide monomer and the styrene monomer, respectively. We also compared measured mass values with simulated theoretical mass values (DP = 9 for NB-PLLA and 15 for PS), confirming norbornene-terminated MMs (Figures S3b and S4b).

### 2.2. Sequential ROMP of Macromonomers

**Figure 1:**
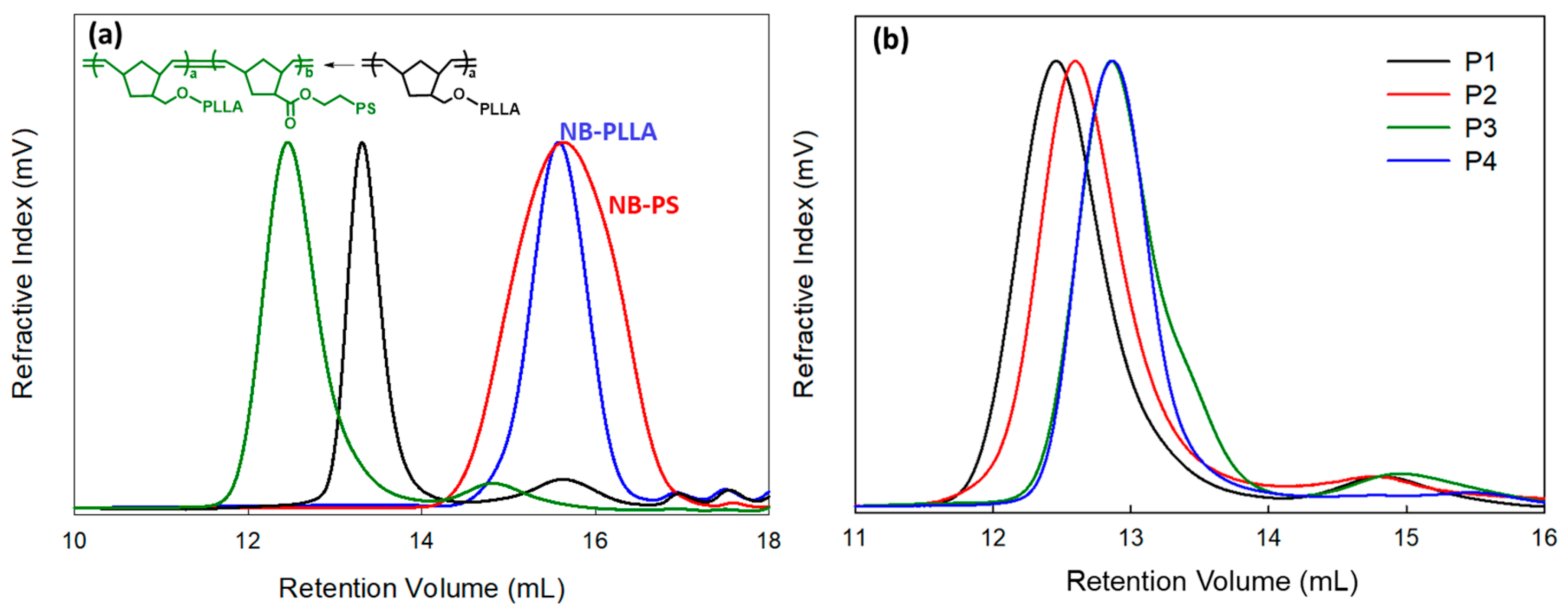
SEC traces: (**a**) NB-PLLA_3.68k_ (blue trace), NB-PS_3.28k_ (red trace), the first block brush homopolymer (black trace), and the final BBCP (P1) (green trace) and (**b**) BBCPs with various PLLA volume fractions.

PLLA-*b*-PS BBCPs with various PLLA volume fractions (*f*_PLLA_) were synthesized by adjusting the feed ratio of PS to PLLA. Similar sized PLLA and PS MMs (M_n_ ~ 3.0 kDa) were used to obtain equivalent radius of effective monomer units, $r_{0}$, for densely grafted bottlebrush architecture (*i.e.,* ${\left(\frac{r_{0,PLLA}}{r_{0,PS}}\right)}^{1/2}≃1$) [15]. BBCPs with relatively narrow molecular weight distributions (Ð = 1.14–1.28) were obtained through sequential ROMP in CH_2_Cl_2_ using NB-PLLA as the first MM in the presence of Grubbs third-generation catalyst. The molecular characteristics of BBCPs are summarized in Table 2, and their SEC traces are shown in Figure 1. Unreacted MMs were successfully removed from the final product by precipitating the crude polymer solution in hexane and washing with acetonitrile, but lower molecular weight species (M_n_ ~ 6 kDa, shoulder and tailing in SEC traces) were hard to remove. The small amount of these impurities (less than 10%) is expected not to affect the self-assembly of BBCPs [23]. The successful sequential ROMP of these two MMs was further confirmed by ^1^H NMR spectroscopy (Figure S5): the norbornenyl alkene protons of MMs around δ 6 ppm disappeared, and the alkene protons of poly(norbornene) backbone appeared around δ 5 ppm. The volume ratio of PLLA to PS blocks was calculated by comparing the integrals of characteristic signals of PLLA and PS side chains using tabulated density values, 1.25 and 1.04 g/cm^3^ for PLLA and PS, respectively [11]. The molecular weights of BBCPs were obtained using SEC with the universal calibration technique by measuring the intrinsic viscosity in order to determine accurate molecular weights regardless of the standards employed in the calibration [33]. The obtained molecular weights are in good agreement with the calculated values.

The thermal properties of BBCPs were examined by TGA and DSC. As shown in Figure S6 for P3 (*f*_PLLA_ = 0.47), a characteristic TGA curve exhibits two distinct stages of weight loss: 200–300 °C for PLLA and 350–450 °C for PS. The DSC analysis of BBCPs was performed after erasing the thermal history at 180 °C during 120 s under the flow of nitrogen gas at 10 °C/min. In the DSC second heating scans (Figure S7a), the glass transition temperatures (T_g_) of PLLA (T_g_ ≈ 60 °C) and PS (T_g_ ≈ 90 °C) were observed from all polymers. However, no hint of PLLA crystallites melting could be observed on the various DSC traces, supporting a poor crystalline arrangement. Interestingly, no recrystallization events were observed for all BBCPs during the subsequent cooling scans, indicating that the crystallization of the PLLA blocks was strongly inhibited (Figure S7b), presumably from the competitive segregation process between the PLLA and PS domains. Besides, the two distinct glass transition events could still be observed for all BBCPs, confirming the phase separation between PLLA and PS domains.

### 2.3. Thin Film Self-Assembly of BBCPs

The selection of solvents for deposition and solvent vapor annealing (SVA) plays an important role in the final morphology of linear PS-*b*-PLA. Based on previously reported findings on PS-*b*-PLA BCPs [34,35,36,37,38,39], we selected chlorobenzene, as deposition solvent for our studies. Chlorobenzene is defined as PS selective solvents (χ_PS-solvent_ < 0.5) based on Hildebrand calculations and the low vapor pressure of chlorobenzene have been shown to drive the formation of well-defined microstructures due to its low evaporation rate during the spin coating process [39,40]. PLLA-*b*-PS BBCPs thin-films (30–50 nm in thickness) were deposited from chlorobenzene solution on Si substrates by spin-coating.

**Figure 2:**
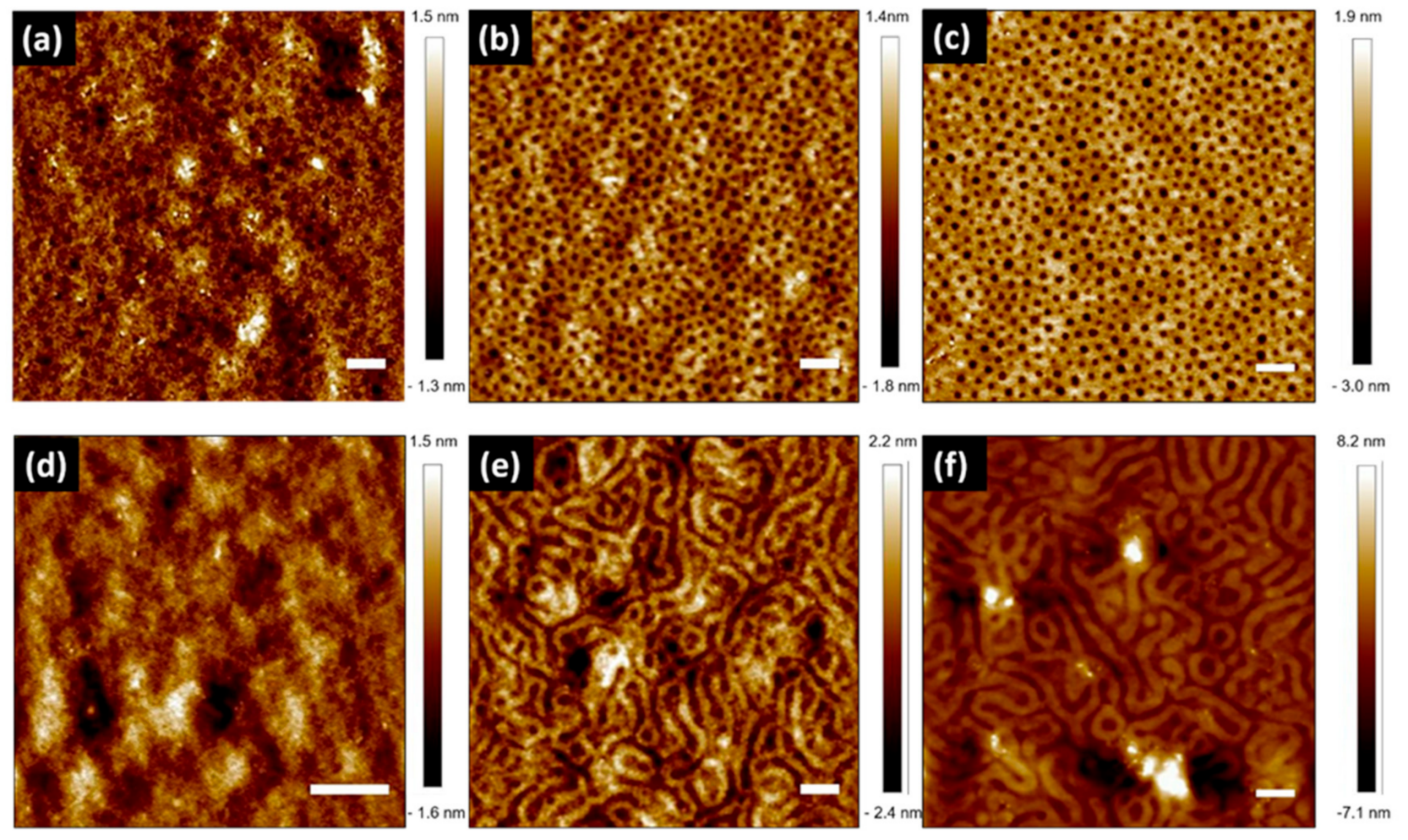
AFM height images of **P1** (**a**–**c**) and **P3** (**d**–**f**) spin-cast from chlorobenzene solution onto Si substrate. (**a**,**d**) as-cast with spin-coating at room temperature; (**b**,**e**) as-cast with spin-coating on heated Si substrate; (**c**,**f**) annealed in chloroform vapor for 5 min after spin-coating on heated Si substrate. Scale bars are 200 nm.

Interestingly, as-cast thin films of PLLA-*b*-PS BBCPs already exhibit self-assembled defective patterns as shown in Figure 2a,d. In order to improve the quality of thin-film morphologies, spin-coating was carried out on heated Si substrates. In fact, it has been demonstrated that spin coating at the temperature above T_g_ of one block and below T_g_ of another block reduce substrate influences on microphase separation of linear BCPs [39]. Prior to spin-coating of PLLA-*b*-PS BBCPs, Si substrates were thus heated at ~75 °C (slightly above T_g_ of PLLA and below T_g_ of PS) for 5 min. We observed that spin-coating on heated Si substrates provided a way to promote ordering and well-defined patterns as shown in Figure 2b,e, respectively. The surface topographies of both PLLA perpendicular cylinders and lamellae are more distinct in comparison to Figure 2a,d. We believe that the escalated substrate temperature increases the evaporation rate of the solvent, thereby driving phase segregation. Moreover, we cannot discard the possibility that the overall mobility of the system was increased due to the elevated substrate temperature. We observed similar behavior after heating substrates at 100 °C (data not shown). However, higher substrate temperature treatment, e.g., >125 °C, resulted in dewetted films.

In order to further enhance pattern definition, we treated as-cast heated films with SVA. The segregation between PS and PLA is affected by solvent uptake, and an effective interaction parameter (χ_eff_) can be defined to relate the influence of the solvent: χ_eff_ = χ(1 − Φ_s_), where χ_eff_ and χ are the Flory-Huggins interaction parameters in the presence and absence of the solvent respectively, and Φ_s_ is the volume fraction of solvent [37]. While the solvent uptake by the PLLA-*b*-PS BBCP films reduces the overall χ, solvent molecules permeating through the films also decrease the T_g_ values of both blocks. SVA treatment under chloroform vapors for as short as 5 min resulted in enhanced long-range order of both morphologies (Figure 2c,f). P1 exhibits a dot pattern possessing a period of 72 nm with domain sizes of 46 nm, while P3 shows a line pattern having period of 102 nm with domain sizes of 58 nm. The obtained period sizes are well beyond those typically accessible by linear BCPs, and achieved in a short period of time. We also investigated the self-assembly of PLLA-rich BBCP (P4, *f*_PLLA_ = 0.75) (Figure S9). Owing to the drastic change in *f*_PLLA_, well-defined patterns were not observed from as-cast or as-cast heated films of P4. However, partial dot features with large variation in domain size were observed after 5 min SVA, showing PS domains within the major component PLLA matrix. In summary, with the increase of the PLLA volume fraction, morphological transitions from PLLA cylinders to lamellae to PS cylinders were observed in accordance with the theoretical phase diagram, recently reported by Zhulina et al. [15].

### 2.4. Solvent Effects on PLLA-b-PS BBCP Thin Film Self-Assembly

**Figure 3:**
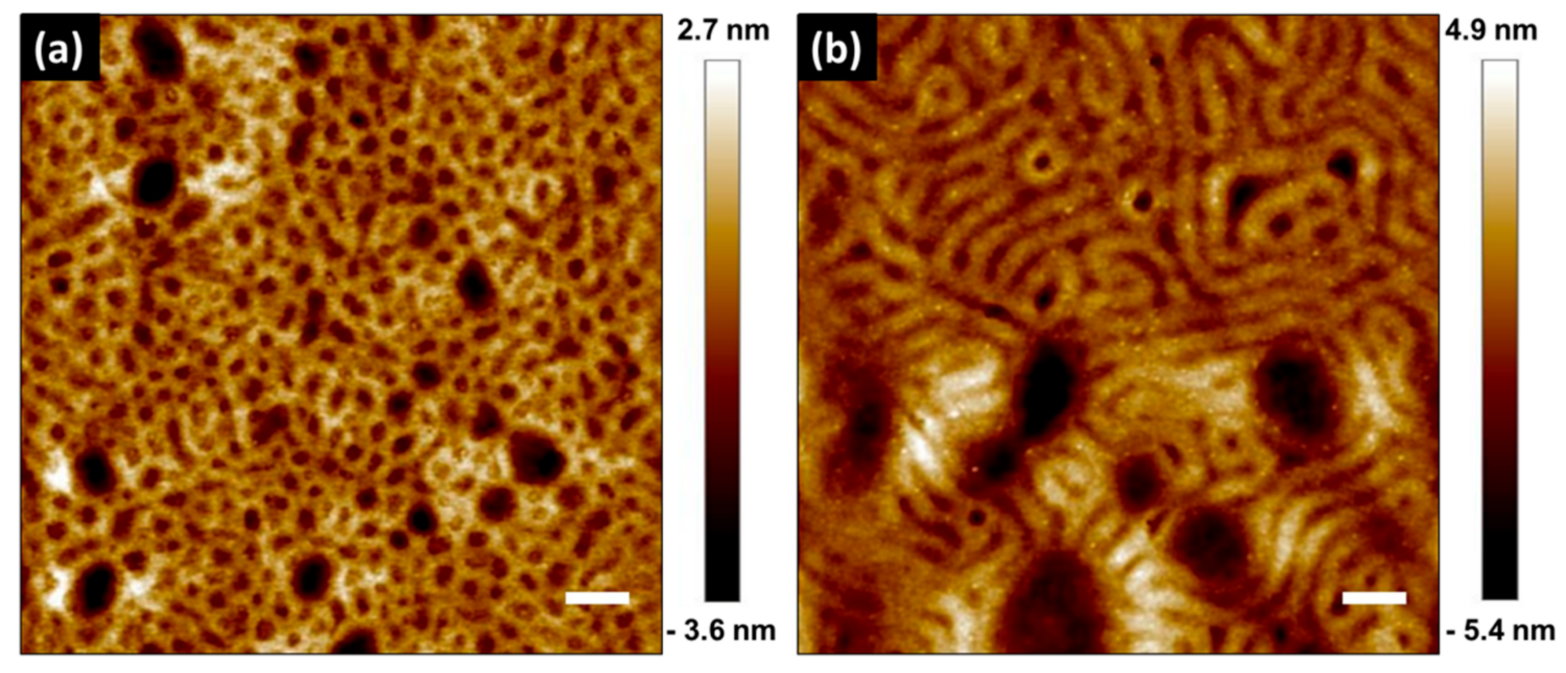
AFM height images of **P2** after spin-coating from THF solution on heated Si substrate followed by solvent vapor annealing in (**a**) THF and (**b**) chloroform for 5 min. Scale bars are 200 nm.

Next, we discuss the polymer-solubility consideration for a cylindrical PLLA-*b*-PS BBCP and the ability to change cylinder orientation depending on SVA conditions. While P1 (*f*_PLLA_ = 0.28) and P3 (*f*_PLLA_ = 0.47) formed cylindrical and lamellar patterns by spin-casting using chlorobenzene, we observed cloudy, highly viscous solutions from P2 (*f*_PLLA_ = 0.37) solution in chlorobenzene, and spin-cast P2 films from chlorobenzene solution on either non-heated or heated substrates did not exhibit a clear microphase separation (Figure S8). In order to rectify this, we used THF as a casting solvent for P2 and a clear solution was readily formed, inferring PLLA and PS miscibility. As shown in Figure 3, P2 shows the ability to orient domain features through either SVA in THF for perpendicular orientation or SVA in chloroform for parallel orientation. This can be explained by the choice of selective solvents and their subsequent effect on the polymer/air interface. Empirical studies have shown that a neutral solvent like THF produces perpendicularly orientated PLA cylinders [30,35]. In contrast, a selective solvent like chloroform orients cylinders parallel to form line-space patterns [41]. Overall, the PLLA-*b*-PS BBCPs developed here show that it is possible to pattern large periodic dot and line features in a rapid timeframe. Moreover, we have demonstrated that the orientation of PLLA cylinders can be controlled to produce perpendicular or parallel features based on the choice of SVA solvent.

### 2.5. Generation of Nanoporous Thin-Films

**Figure 4:**
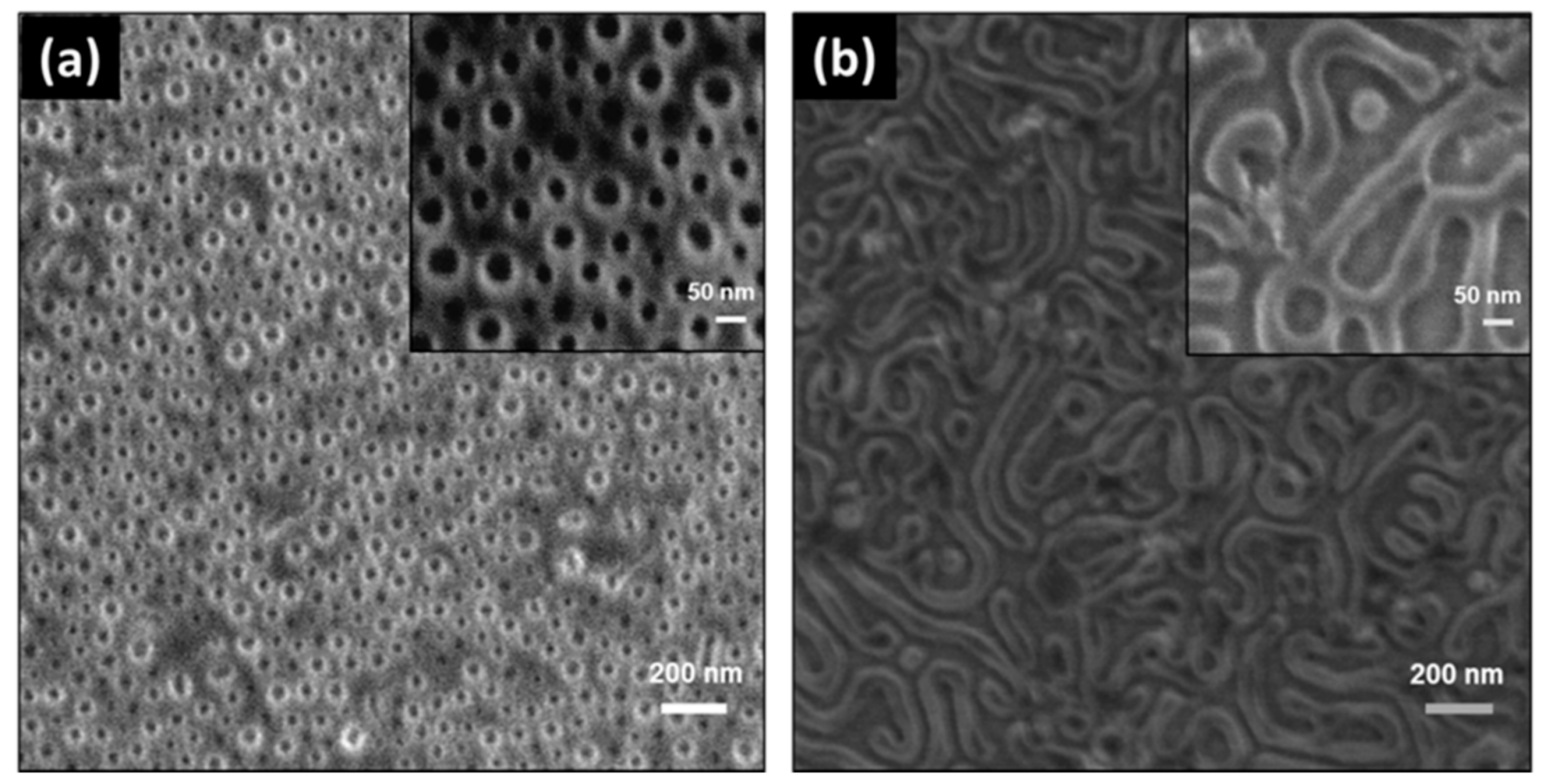
Top-down SEM images of **P1** (**a**) and **P 3** (**b**) after oxygen plasma etching.

BBCPs containing PLA are highly attractive materials for the fabrication of nonporous materials because of the degradable ester group which can be hydrolytically decomposed. The dry etching of PLA has been known to produce a good-quality pattern over a large area compared to wet etch methods [42]. Thus, we used oxygen plasma treatment to selectively remove the PLLA domains, which are etched faster than the PS domains under O_2_ chemistry due to the susceptibility of the PLA ester linkages [43,44]. Figure 4a,b displays top-down SEM images of PLLA-*b*-PS BBCPs films after a brief oxygen plasma treatment, revealing well-defined dot and line-space patterns. The obtained porous PS film template are ideal to be used for pore-filling with a variety of ingredients such as metals or dielectrics for membranes, photonics, and catalysis applications [45,46,47].

## 3. Conclusions

In summary, we prepared a series of well-defined PLLA-*b*-PS BBCPs, and investigated their morphologies as a function of the relative block volume factions. Interestingly, BBCPs containing similar sized side chains self-assembled rapidly into ordered cylindrical or lamellar structures. By simply varying *f*_PLLA_ from 0.27 to 0.75, morphological transitions occurred (cylinder-lamellae-cylinder). For P1 and P3, cylindrical and lamellar morphologies were observed right after spin-coating, and P2 showed the change in orientation of cylindrical domains depending on the type of solvent for SVA for 5 min. We initially expected the PLLA crystallinity to enrich the self-assembly behavior of PLLA-*b*-PS BBCPs, possibly leading to the volume fraction dependent morphology transitions like linear BCPs. Further studies to leverage the crystallinity of PLLA as an additional tool to tune BBCP self-assembly are required in the future to fully comprehend the mechanism in these systems. Additionally, PLLA-*b*-PS BBCPs films were used to prepare nonporous templates by oxygen plasma etching. We believe that the facile synthesis of PLLA-PS BBCP disclosed here contribute to a better understanding of the relationship between BBCP architecture and the enhanced self-assembly behavior in thin film form. The findings progress our knowledge on the growing interest of controlling BBCPs in thin-films to produce functional materials.

## Figures and Tables

**Table 1:** Molecular characteristics of NB-PLLA and NB-PS MMs.

	*M_n_*_,SEC_ (Da) ^a^	Ð ^a^	*M_n,_*_NMR_ (Da) ^b^	DP_NMR_^b^	*M_n_*_,MALDI_ (Da) ^c^	Ð ^c^
**NB-PLLA_2.58_**	2580	1.08	1850	12	-	-
**NB-PLLA_3.68k_**	3680	1.16	2570	17	2660	1.05
**NB-PS_3.28k_**	3280	1.40	3450	31	3260	1.10

^a^ Determined by SEC in THF using refractive index detector with PS standards. ^b^ Calculated by comparing integrations of PS and PLLA proton signals from^1^H NMR spectra in CD_2_Cl_2_. ^c^ Determined by MALDI-TOF MS (linear mode).

**Table 2:** Characteristics of PLLA-*b*-PS BBCPs synthesized and studied in this work.

	MM_1_^a^	MM_2_^a^	[MM_1_/C]: [MM_2_/C]	*M_n_* (kDa) ^b^	Ð ^a^	*f*_PLLA_^c^	Domain Size (nm) ^d^	Period (nm) ^d^	Morphology ^d^
**P1**	PLLA_3.68k_	PS_3.28k_	30:75	301	1.25	0.28	46	72	PS C_⊥_
**P2**	PLLA_3.68k_	PS_3.28k_	30:60	275	1.28	0.37	51	92	PS C_∥_
**P3**	PLLA_2.56k_	PS_3.28k_	30:30	171	1.20	0.47	58	102	L_⊥_
**P4**	PLLA_3.68k_	PS_3.28k_	30:15	220	1.14	0.75	-	70	PLLA C_⊥_

^a^ Determined by SEC in THF using refractive index detector with PS standards. ^b^ Determined by SEC in THF using the universal calibration technique. ^c^ Calculated by comparing integrations of PS and PLLA proton signals from ^1^H NMR spectra in CD_2_Cl_2_. ^d^ Determined by AFM image analysis (C_⊥_: perpendicular cylinders; C_∥_: parallel cylinders; L_⊥_: perpendicular lamellae).

## Data Availability

The data presented in this study are available on request from the corresponding author.

## Supplementary Materials

The following are available online, Experimental section, Figure S1: ^1^H NMR and ^13^C NMR Spectra of NB-PLLA, Figure S2: ^1^H NMR and ^13^C NMR Spectra of NB-PS, Figure S3: MALDI-TOF MS spectrum of NB-PLLA, Figure S4: MALDI-TOF MS spectrum of NB-PS, Figure S5: 1H NMR spectra of two MMs and of the resulting BBCP, Figure S6: TGA of P3 under nitrogen atmosphere, Figure S7: DSC thermograms of BBCPs recorded at heating rate of 10 °C/ min under nitrogen, Figure S8: AFM height images of P2 spin-cast from chlorobenzene solution onto Si substrate, Figure S9: AFM height images of P4 spin-cast from chlorobenzene solution onto Si substrate. Experimental details and additional characterization data (^1^H NMR and ^13^C NMR spectra, MALDI-TOF spectra, DSC and TGA thermograms, and AFM images).
