# Supplementary material for: Precise Synthesis and Thin Film Self-Assembly of PLLA-b-PS Bottlebrush Block Copolymers
Source: Molecules. 2021 Mar 5;26(5):1412. doi: 10.3390/molecules26051412 (PMC7961899; doi:10.3390/molecules26051412)
Supplement: Supplementary file 1 [file molecules-26-01412-s001.pdf]

# Supporting Information

# Precise Synthesis and Thin Film Self-Assembly of PLLA-*b*-PS Bottlebrush Block Copolymers

*Eunkyung Ji‡, Cian Cummins‡, Guillaume Fleury**

Laboratoire de Chimie des Polymères Organiques, Université de Bordeaux, CNRS, Bordeaux INP, LCPO, UMR 5629, F-33600 Pessac, France

## EXPERIMENTAL SECTION

**Materials.** All chemicals used for synthesis were obtained from commercial sources and used as received unless otherwise noted. THF, toluene, and styrene were purified as described previously.[1] L-lactide was recrystallized from ethyl acetate twice, and kept under vacuum prior to use. $CH_2Cl_2$ was obtained from the mBRAUN solvent purification system. All reactions were carried bout in flame-dried Schlenk-type glassware on a dual-manifold Schlenk line or in an argon-filled glove box.

**Characterization.** $^1$H NMR and $^{13}$ NMR spectra were recorded with a Bruker 400 MHz NMR spectrometer and chemical shifts were reported in ppm using deuterated solvents as internal standards ($CD_2Cl_2$, 5.26 ppm and $CDCl_3$, 7.27 ppm). Size exclusion chromatography (SEC) was performed by using a Viscotek TDAmax system that consists of the GPCmax integrated solvent

and sample delivery module, two Agilent columns (PLgel 5 μm MIXED-C, 300 x 7.5 mm), a guard column (PL gel 5 μm), and a Tetra Detector Array (TDA) including a light scattering (LS) detector (RALS/LALS), a four-capillary differential viscometer, a differential refractive index detector (RI), and a diode array UV detector. THF was used as a mobile phase with a flow rate at 0.8 mL/min at 30 °C.

Matrix-assisted laser desorption/ionization time-of-flight mass spectrometry (MALDI-TOF MS) analysis were performed using the Bruker autoflex maX equipped with a frequency tripled Nd:YAG laser emitting at 355 nm. Mass spectra were acquired in the positive-ion reflectron and linear mode with an accelerating voltage of 19 kV. Samples were dissolved in THF at 10 mg/ml. Dithranol (1,8-dihydroxy-9,10-dihydroanthracen-9-one) and DCTB (trans-2-[3-(4-tert-Butylphenyl)-2-methyl-2-propenylidene]malononitrile) matrix solutions were prepared by dissolving 10 mg in 1 ml of THF for NB-PS and NB-PLLA, respectively. Cationization agent solutions (AgTFA in THF for NB-PS and NaI in MeOH for PLLA, 10 mg/ml) were also prepared. All three solutions were combined in a 10:1:1 volume ratio (matrix: sample: cationization agent). One to two microliters of the obtained solution was deposited onto the sample target and dried under vacuum.

Thermogravimetric analysis (TGA) was recorded using a TA instruments Q 50 in the range of from 20 to 580 °C with heating rate of 10 °C/ min under nitrogen atmosphere. Differential scanning calorimetry (DSC) was performed on TA instruments Q 100 equipped with a refrigerated cooling system under nitrogen atmosphere. Firstly, the samples were held at 180 °C for 2 min in order to erase previous thermal history. DSC traces were then recorded from the subsequent heating and cooling scans at a rate of 10 °C/ min.

AFM (Dimension Fast Scan, Bruker) was used in tapping mode to characterize the surface morphology of BBCPs and silicon cantilevers (Fastscan-A) with a nominal tip radius of 5 nm and a spring constant about 18 N $m^{-1}$ were used. The resonance frequency of the cantilevers was about 1400 kHz. A JEOL 7800-E Prime SEM was used in super high-resolution gentle beam (GBSH) with an accelerating voltage of 1kV.

**Synthesis of PLLA macromonomers**. PLLA macromonomers were synthesized using ring opening polymerization (ROP) of L-lactide using *exo*-5-norbornene-2-methanol as an initiator and $Sn(Oct)_2$ as a catalyst. *exo*-5-norbornene-2-methanol was synthesized according to the previously described procedures.[2] Under an argon atmosphere, L-lactide (3.43 g, 23.81 mmol) and *exo*-5-norbornene-2-methanol (0.15 g, 1.19 mmol) were added into a flame-dried Schlenk flask connected to the toluene filled flask. After the Schlenk flask was evacuated and backfilled with argon three times, 40 mL of toluene was introduced to dissolve the monomer. Then, the flask was placed in an oil bath heated to 60 °C, and polymerization was initiated by the addition of $Sn(Oct)_2$ (1M in toluene, 1.19 mL, 1.19 mmol) via a syringe. After 18h, the resulting solution was cooled to room temperature and an aliquot of the resulting solution was taken for conversion analysis by $^1$H NMR prior to precipitation into cold methanol. The obtained polymer (98% conversion) was filtrated and washed with methanol, and dried in a vacuum oven overnight.

**Synthesis of PS macromonomers.** The desired ω-norbornenyl polystyrene (NB-PS) was synthesized by living anionic polymerization of styrene followed by adding ethylene oxide and end-capping with *exo*-5-norbornene-2-carbonyl chloride (*exo*-NB-COCl).[3] Since *exo*-NB-COCl was not commercially available, it was prepared by chlorination reaction of *exo*-5-norbornene-2-carboxylic acid with oxalyl chloride and cat. DMF in $CH_2Cl_2$. After solvent was removed, synthesized *exo*-NB-COCl was diluted in THF to the desired concentration and kept

inside a refrigerator without additional purification, and used for *in-situ* termination reaction during the anionic polymerization of styrene. The anionic polymerization was carried out in THF at -78 $^{o}$C for 40 min after the addition of styrene to the mixture of *sec*-BuLi and THF. After addition of ethylene oxide to the polymerization mixture at -50 $^{o}$C, the mixture solution was slowly warmed to room temperature overnight. Then, *exo*-NB-COCl solution in THF was added for end-functionalization, and the mixture solution was stirred at room temperature for 1 day under argon atmosphere. To improve end-capping efficiency of MMs, excess *exo*-NB-COCl ([NB-COCl]/ [*sec*-BuLi] = 4) was used. The resulting polymer was isolated by precipitation in methanol, filtration, and drying under vacuum.

**Sequential ROMP procedures for BBCPs.** Macromonomers was kept in vacuum oven prior to use. Desired amount of NB-PLLA and NB-PS were added into a separate flame-dried Schlenk flask equipped with a stir bar, and the flasks were evacuated and backfilled with argon three times. In a glove box, two polymer solution and a Grubbs catalyst (G3) stock solution (1mg/ 0.15 mL) were prepared in $CH_2Cl_2$. ROMP polymerization was initiated by adding the required volume of the catalyst solution ([MM]/[G3] = degree of polymerization) to the first macromonomer (NB-PLLA) solution. The reaction was allowed to run for 90 min and then an aliquot was withdrawn for SEC analysis before adding the second macromonome solution. The aliquot was quenched with ethyl vinyl ether for 30 min. The mixture solution was kept stirring for another 3 hr, and quenched with ethyl vinyl ether for 30 min. The resulting polymer was precipitated into methanol and then hexane followed by washing with acetonitrile. Depending on the PLLA volume fraction, washing with acetonitrile is not necessary. For example, for BL16 ($f_{PLLA}$ = 0. 75), a clean product was obtained after precipitation in hexane and washing with hexane.

**Preparation of thin-films**

Planar Si substrates (from Si-Mat silicon materials) used were highly polished single-crystal silicon <100> wafers (p-type) with a native oxide layer of ~2 nm. Si wafers were initially cleaned using organic solvent (chloroform) by ultra sonication to degrease substrates and remove any contaminants. After ultra sonication, Si wafers were treated with UV/$O_3$ for 10 mins to remove organic contaminants. PLLA-PS BBCP solutions were dissolved in chlorobenzene (for **P1**, **P3** and **P4**) and THF (for **P2**) and left stirring until fully dissolved displaying clear solutions. 0.85 μLof respective solutions were then deposited on UV/$O_3$ cleaned Si surfaces. For the deposition on heated Si wafers, wafers were pre-heated at 75°C on a hot plate and then immediately coated with respective PLLA-PS BBCP solution. For solvent vapor annealing studies, films were placed in a glass jar and left for the desired time with a small vial of the anneal solvent (THF or chloroform).

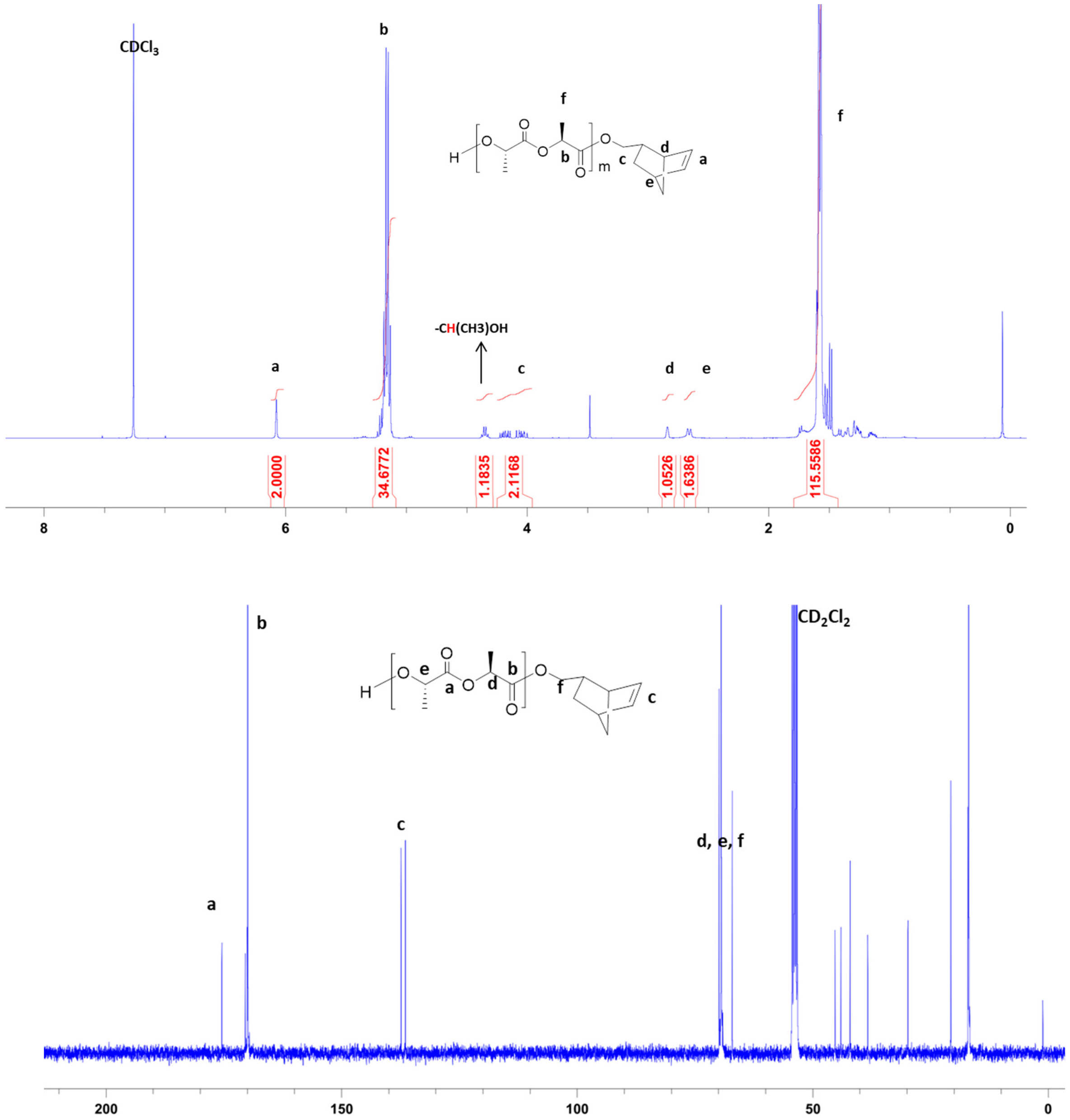


**Figure S1**. $^{1}$H NMR and $^{13}$C NMR Spectra of NB-PLLA in $CDCl_3$ and $CD_2Cl_2$, respectively.

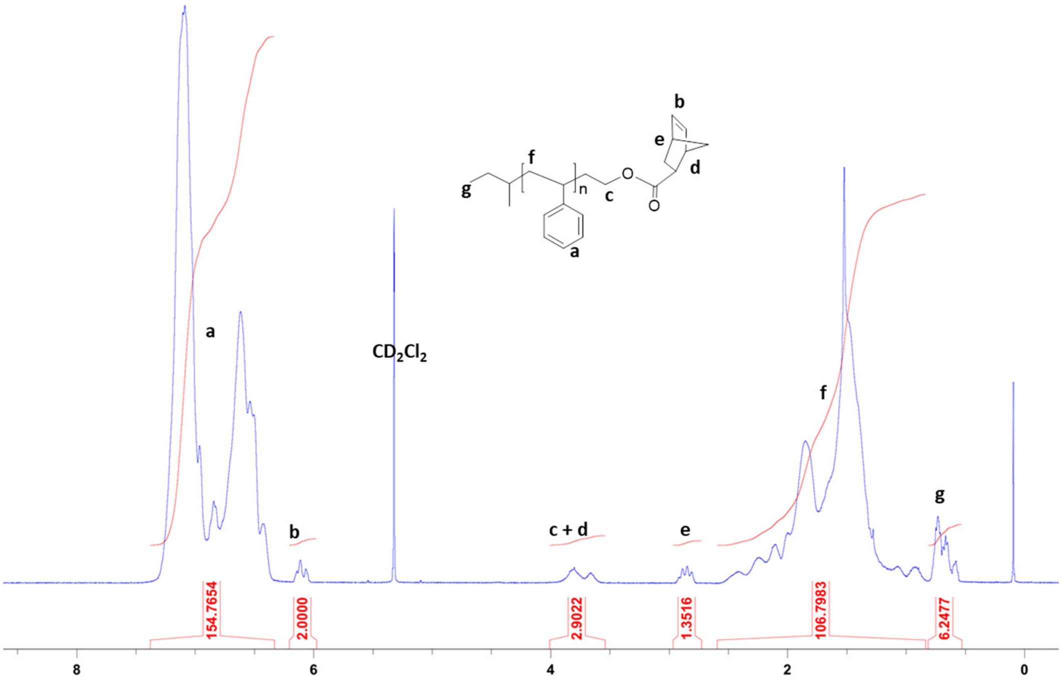


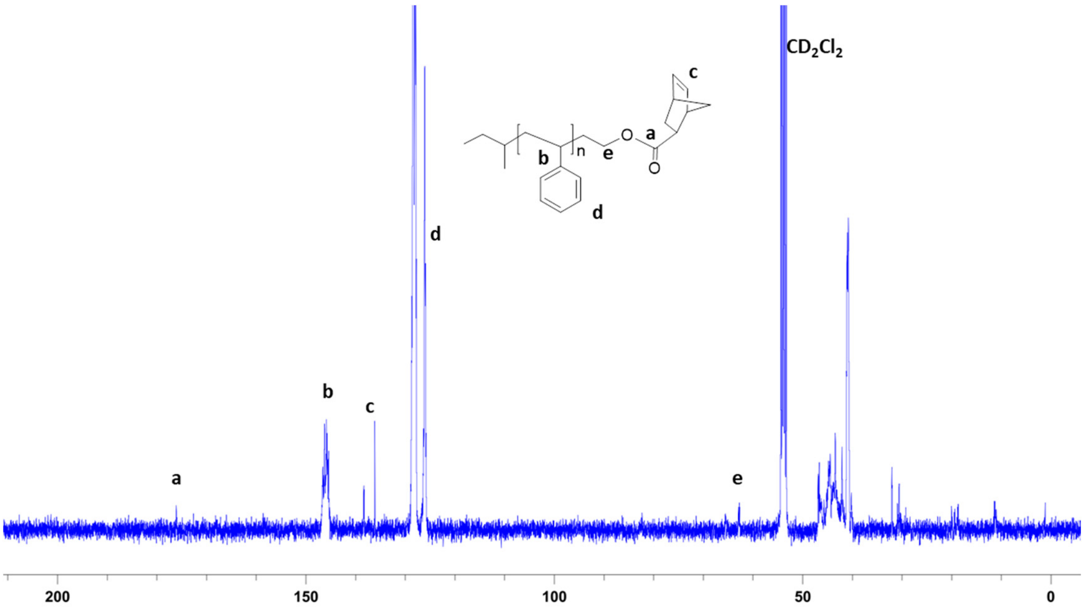


**Figure S2**. $^{1}H$ NMR and $^{13}C$ NMR Spectra of NB-PS in $CD_2Cl_2$

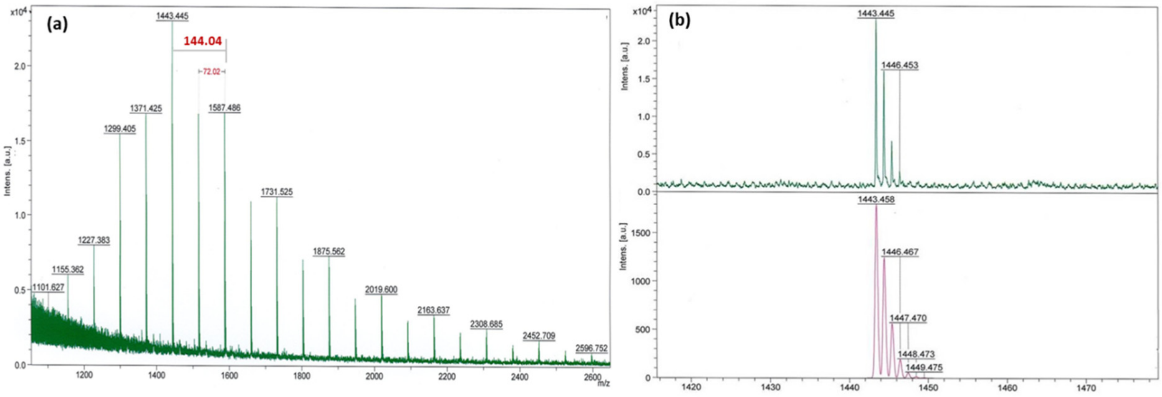


**Figure S3**. MALDI-TOF MS spectrum of NB-PLLA (a) and comparison of raw data with simulated data (b).

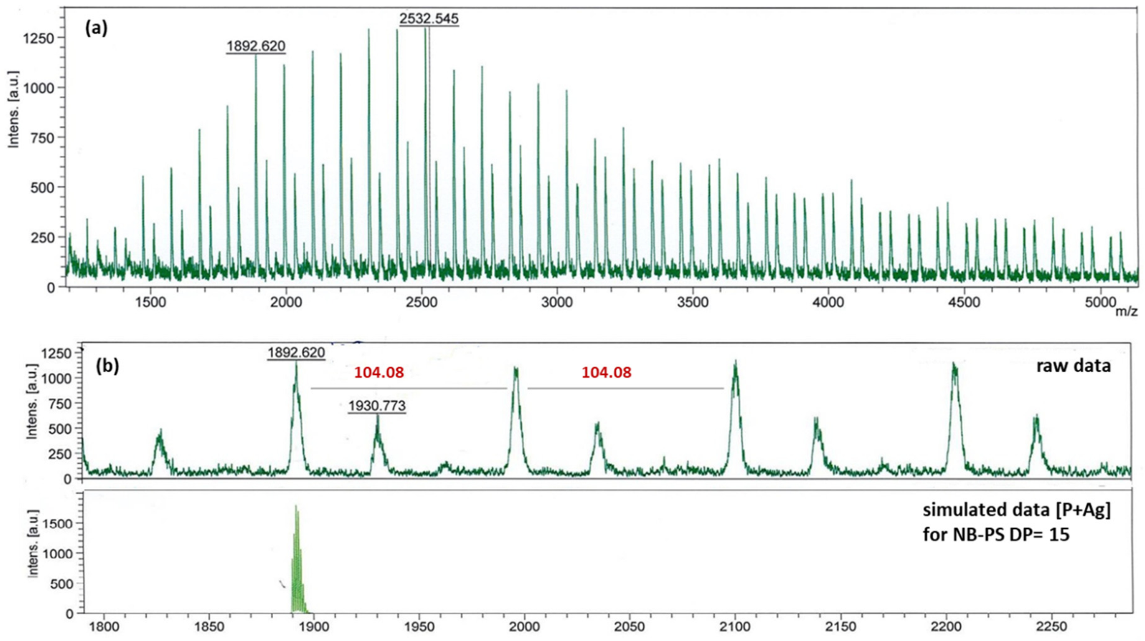


**Figure S4**. MALDI-TOF MS spectrum of NB-PS (a) and comparison of raw data with simulated data (b).

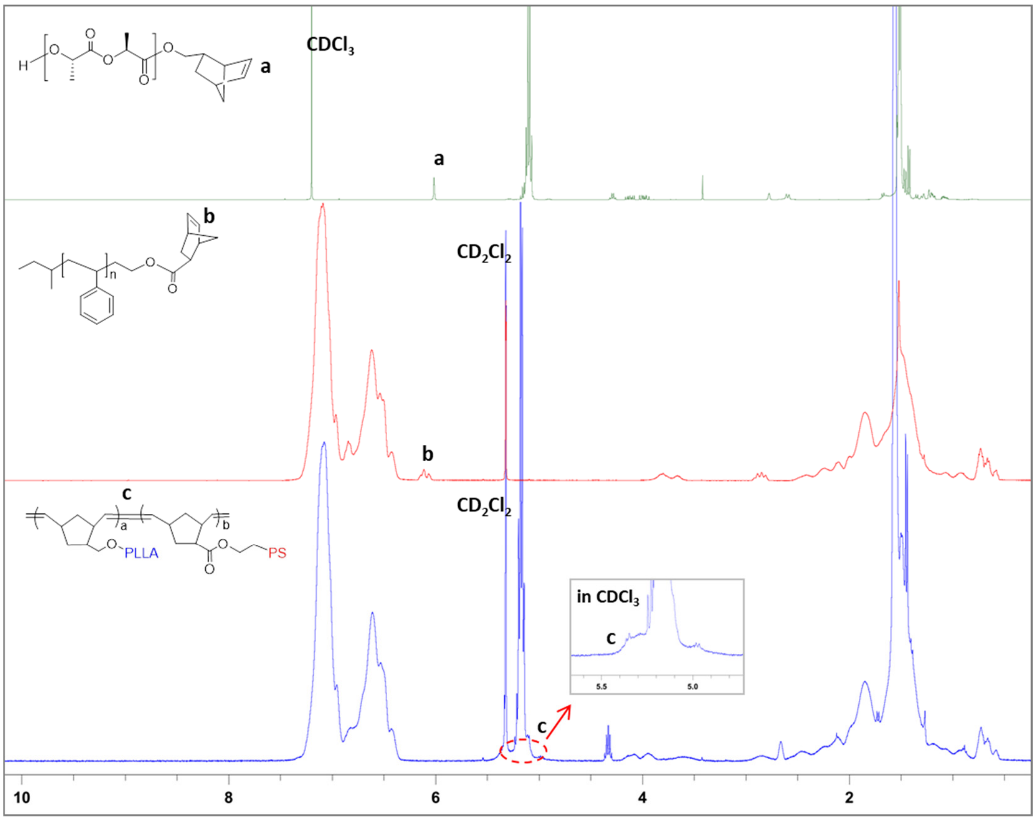


**Figure S5.** $^{1}$H NMR spectra of two macromonomers and of the resulting BBCP: (a) NB-PLLA, (b) NB-PS and (c) BBCP.

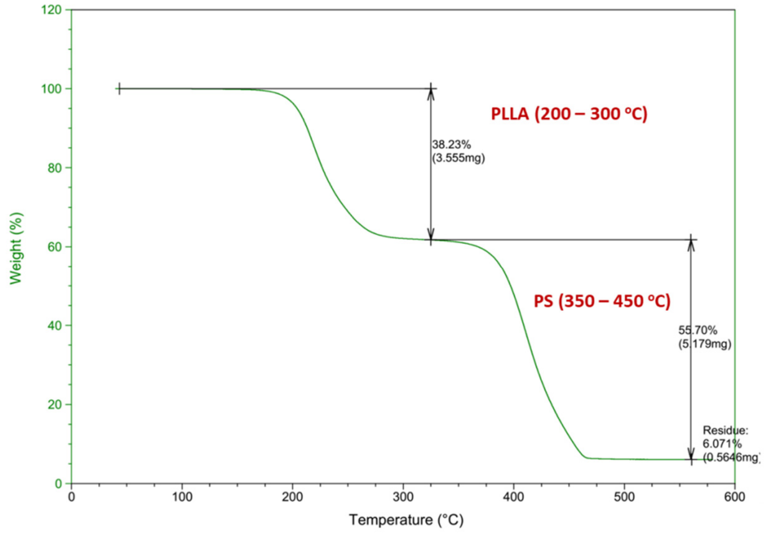


**Figure S6.** TGA of **P3** under nitrogen atmosphere.

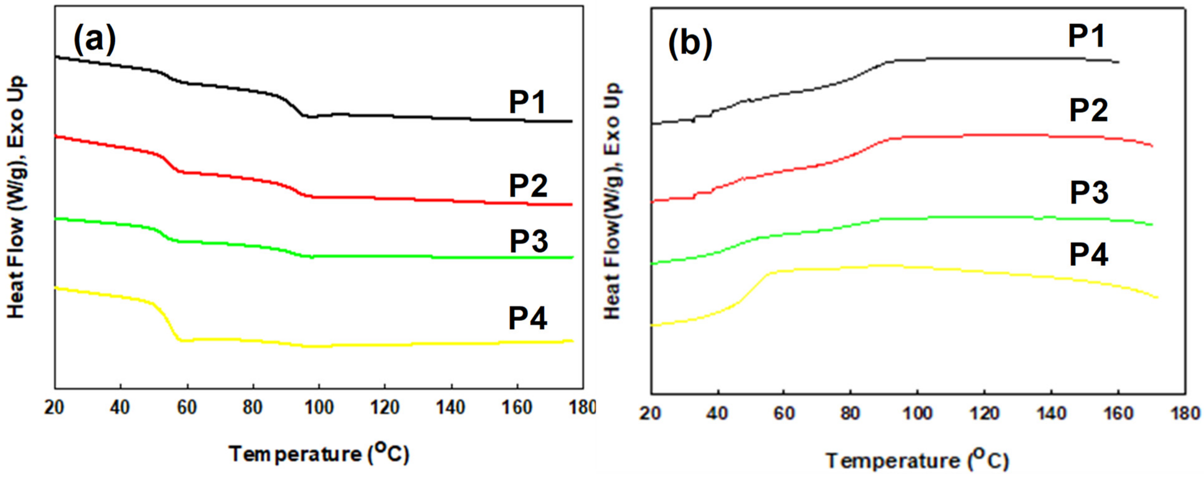


**Figure S7**. DSC thermograms of BBCPs recorded at heating rate of 10 ºC/ min under nitrogen: (a) the second heating; (b) and the subsequent cooling.

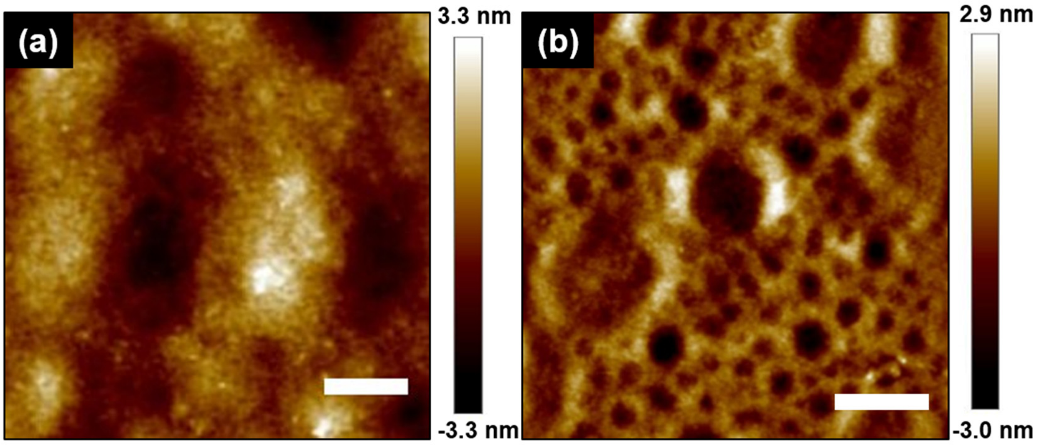


**Figure S8**. AFM height images of **P2** ($f_{\mathrm{PLLA}}$ = 0.37) spin-cast from chlorobenzene solution onto Si substrate. (a) as-cast with spin-coating at room temperature; (b) as-cast with spin-coating on heated Si substrate. Scale bars in all images are 200 nm.

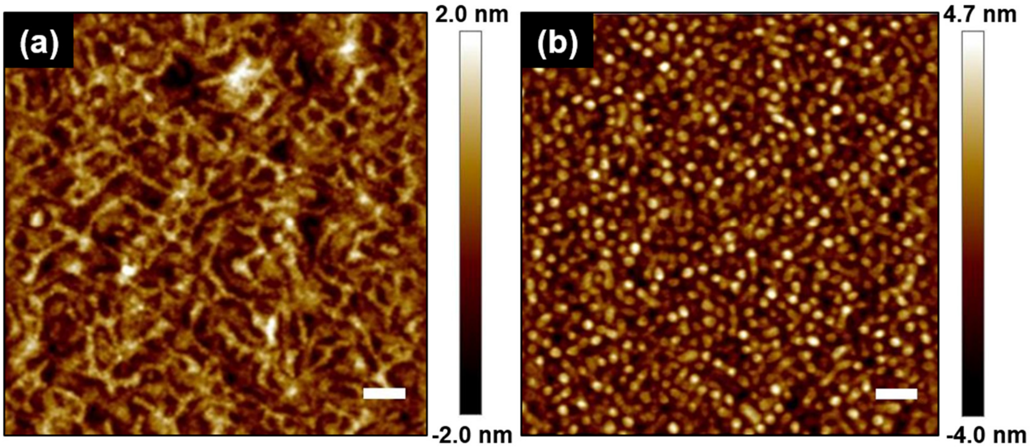


**Figure S9**. AFM height images of **P4** ($f_{\mathrm{PLLA}}$ = 0.75) spin-cast from chlorobenzene solution onto Si substrate. (a) as-cast with spin-coating on heated Si substrate; (b) annealed in chloroform vapor for 5 min after spin-coating on heated Si substrate. Scale bars in all images are 200 nm.

## REFERENCES


1. Hadjichristidis, N.; Hirao, A. *Anionic Polymerization: Principles, Practice, Strength, Consequences and Applications*; Hadjichristidis, N., Hirao, A., Eds.; Springer Japan: Tokyo, 2015; ISBN 9784431541868.
2. Zha, Y.; Disabb-Miller, M.L.; Johnson, Z.D.; Hickner, M.A.; Tew, G.N. Metal-cation-based anion exchange membranes. *J. Am. Chem. Soc.* **2012**, *134*, 4493–4496, doi:10.1021/ja211365r.
3. Theodosopoulos, G. V.; Bitsi, S.L.; Pitsikalis, M. Complex Brush-Like Macromolecular Architectures via Anionic and Ring Opening Metathesis Polymerization: Synthesis, Characterization, and Thermal Properties. *Macromol. Chem. Phys.* **2018**, *219*, 1700253, doi:10.1002/macp.201700253.
